# Physical activity after revision knee arthroplasty including return to sport and work: a systematic review and meta-analysis including GRADE

**DOI:** 10.1186/s12891-023-06458-y

**Published:** 2023-05-09

**Authors:** Sten van der Wilk, Alexander Hoorntje, Leendert Blankevoort, Rutger van Geenen, Gino M. M. J. Kerkhoffs, P. Paul F. M. Kuijer

**Affiliations:** 1 Department of Orthopaedic Surgery & Sports Medicine, Amsterdam UMC, University of Amsterdam, Amsterdam Movement Sciences, Amsterdam, The Netherlands; 2 Amsterdam Movement Sciences, Program Musculoskeletal Health, Amsterdam, The Netherlands; 3 Department of Orthopaedic Surgery, Amphia Hospital, Breda, The Netherlands; 4 Department Public and Occupational Health, Amsterdam UMC, University of Amsterdam, Amsterdam Public Health Research Institute, Amsterdam Movement Sciences, Amsterdam, The Netherlands

**Keywords:** Knee arthroplasty, Knee replacement, Revision, Recovery of function, Physical activity, Return to work, Return to sport, Prognosis, Osteoarthritis

## Abstract

**Background:**

The number of primary knee arthroplasties (KAs) performed annually is rising, especially among active, working age patients. Consequently, revision KA is also increasingly performed. Our aim was to systematically review the extent to which patients were physically active following revision KA, and the rate and timing of return to sport and work.

**Methods:**

A search was conducted in the databases Medline and Embase until February 24^th^, 2023. Studies describing patients with revision total knee arthroplasty (rTKA) or revision unicondylar knee arthroplasty (rUKA), with outcomes regarding physical activity or return to sport (RTS) or work (RTW) were included. Quality of studies was assessed using the Newcastle–Ottawa scale, meta-analyses were performed using RevMan 5.4 and Grading of Recommendations, Assessment, Development, and Evaluations (GRADE).

**Results:**

Of the 4,314 articles screened, 22 studies were included describing 2,462 rTKA patients (no rUKA), 42% were male with a mean age of 67 years (range 24 – 95). No studies reported objective physical activity measurements. Twenty-two studies reported patient reported outcome measures (PROMs). The PROMs that were pooled on a scale from zero to ten were the UCLA Activity Score, the Tegner Activity Level Scale, the Lower-Extremity Activity Scale, Devane Activity Score, and physical activity related subscales of the Knee injury and Osteoarthritis Outcome Score. The retrospective studies of moderate quality showed a statistically significant postoperative improvement of 1.7 points (MD = 1.71, 95% CI 1.48 – 1.94 (*p* < 0.0001); 14 studies, *n* = 1,211). For the prospective moderate-quality studies, a statistically significant postoperative increase of 0.9 points was found (MD = 0.89, 95% CI 0.48 – 1.30 (*p* < 0.0001); 6 studies, *n* = 1,027). Regarding RTS, 12% of patients participated in so-called ‘non-recommended’ activities (i.e., hockey, soccer, football, gymnastics, jogging, singles tennis, and basketball) after rTKA (1 study, *n* = 206). The pooled RTW was 86% (2 studies, range 18–95%, *n* = 234).

**Conclusions:**

The majority of patients self-reported an improved postoperative activity level after rTKA. Patients could maintain an active lifestyle in daily life, including sports and work. For reliable physical activity, RTS and RTW estimations, more studies are required. In terms of GRADE, the quality of evidence for the five prospective studies was rated as low.

**Level of evidence:**

Level 3.

**Supplementary Information:**

The online version contains supplementary material available at 10.1186/s12891-023-06458-y.

## Background

Osteoarthritis (OA) is a degenerative and progressive joint disease affecting more than 25% of the adult population [1, 2]. Multiple factors contribute to the risk of developing OA, including genetic predisposition, sport injuries, physical work overload, obesity, and aging [3]. OA is one of the most common causes of adult disability worldwide. When conservative treatment of OA has failed, surgical intervention may be considered. Knee arthroplasty (KA) is a surgical option for patients with knee OA [4–7], which provides pain relief, restored knee joint function, and improved quality of life [8]. Both unicondylar knee arthroplasty (UKA) and total knee arthroplasty (TKA) are cost-effective methods [4, 9, 10].

The increasing prevalence of knee OA contributes to a higher demand for KA. The number of primary knee arthroplasty (pKA) procedures is increasing and the largest increase is seen in younger patients (< 65 years of age) [11]. A similar increase is expected for revision KA [12, 13]. For example, an 88% increase in revision TKA (rTKA) is expected in Germany by 2050, compared to 2020 [14]. A substantial increase in rTKA is also expected in the United States, with a projected increase between 78 and 182% for 2030, compared to 2014 [15]. Performing more pKAs, particularly in younger patients, increases the likelihood of revision surgery. This can generally be attributed to a more active lifestyle, increased life-expectancy, and the limited lifespan of knee implants [16]. For example, Bayliss et al. found a 35% revision risk for male patients < 55 years of age, with a median time to revision of 4.4 years [17]. Additionally, Walker-Santiago et al. described that early reoperations, early re-revisions, and overall re-revisions were generally more common in patients 55 years or younger when compared to older rTKA patients [18].

Currently, there is a lack of clear insight into possibilities for patients regarding physical activity and maintaining an active lifestyle after rKA. As stated, younger patients are at a higher risk of requiring revision KA [17, 19]. Especially for these younger patients, remaining active and returning to activities such as sports and work is important. However, after revision, an active lifestyle and returning to work seem less likely [20]. Patients need to be well informed before receiving KA, considering that several studies showed that patients tend to overestimate their postoperative outcomes [5, 21, 22].

Therefore, our aim was to conduct a systematic review to assess the extent to which patients were physically active following rKA, as well as the rate and timing of RTS and RTW.

## Methods

For this systematic review, the guidelines of the PRISMA 2020 statement were used, and a non-published study protocol was written before the initiation of the study [6].

### Searches

A clinical librarian developed the search strategy in collaboration with the authors, which was validated using several preselected papers that fulfilled the inclusion criteria. Databases used for identifying relevant literature were Medline via Pubmed and Embase via OvidSP. Searches for relevant literature were performed until February 24^th^, 2023, using the following terms and synonyms: ‘knee arthroplasty’, ‘revision’, ‘recovery of function’, ‘sport’, and ‘work’. The entire search, with all terms and synonyms, used for both Medline and Embase can be found in Additional file 1.

### Study inclusion and exclusion criteria

The inclusion criteria for the study were (1) patients receiving rTKA after pTKA, rUKA after pUKA or rTKA after pUKA; (2) studies concerning physical activity, which included one of the following (post-operative) patient-reported outcome measures (PROMs) of interest: Physical activity measurements (e.g., activity trackers) [23], PROMS regarding physical activity (namely the University of California at Los Angeles (UCLA) activity scale [24], Tegner activity score [25], Knee Injury and Osteoarthritis Outcome Score Physical Function Shortform (KOOS-PS) [26], Knee Injury and Osteoarthritis Outcome Score Function in Sport and Recreation (KOOS-Sport/Rec) [27], Devane activity score [28], and Lower-Extremity Activity Scale (LEAS)) [29]; (3) Studies reporting return to sport (RTS) and/or return to work (RTW) rates. After duplications were removed, all titles and abstracts were reviewed independently by at least two of three reviewers (SvdW, AH, PK), using Rayyan [30]. For the included papers, the full text was obtained and assessed independently for eligibility. Any disagreements were resolved by discussion between the three reviewers. No studies were excluded based on language. Studies with a publication date before the year 2000 were excluded due to a shift in patient demographics and recent advancements in bearing surfaces and component design. Furthermore, all case-studies and systematic reviews were excluded. Additionally, the reference lists of the included studies were manually screened for relevant additional studies.

### Outcome measures

Primary outcomes for the present study were physical activity measures (i.e., activity tracker data), PROMs including the UCLA activity scale (ranging from zero to ten, with zero indicating wholly inactive and ten indicating regular participation in impact sports), Tegner activity score (ranging from zero to ten, with zero indicating disability or sick leave pension due to knee problems and ten indicating national elite sports level), KOOS (ranging from zero to 100, with zero indicating extreme difficulty in function and 100 indicating no difficulty in function), Devane activity score (ranging from one to five, with one indicating participation in contact sports and five indicating sedentary/dependent), and LEAS score (ranging from zero to a maximum of 18, with 18 representing daily participation in sports at a competitive level), and RTS and RTW.

### Data extraction strategy

For data extraction, a standardized form was used, which included the following data: (1) study information, including author, year of publication, country, and language; (2) study design and duration of follow-up; (3) study population characteristics such as the number of patients, age, and sex; (4) type of operation performed; (5) outcome measures used; (6) preoperative score; (7) postoperative score; (8) statistical comparison of pre- and postoperative scores; (9) percentage and timing of RTS; (10) percentage and timing of RTW; (11) confounders included; (12) conclusion. Two authors independently extracted data from the included articles (SvdW, AH, PK), and disagreements were resolved through discussion. When data were unclear or missing, authors were contacted for additional information.

### Study quality assessment

To assess the quality of the included studies, the Newcastle–Ottawa Scale (NOS) was used [31]. The NOS includes three categories for quality assessment: 1. selection (four items, namely representativeness of the exposed cohort, selection of the non-exposed cohort, ascertainment of exposure and demonstration that the outcome of interest was not present at the start of the study), 2. comparability (two items, namely comparability of cohorts, and whether the study accounts for possible confounders like sex, age, BMI, advice given by the surgeon or the patient’s motivation), and 3. outcome (three items, namely assessment of outcome, length of follow-up, and adequacy of follow-up). A total of nine stars can be obtained and eight or more stars were considered as indicating a low risk of bias (high quality), five to seven as indicating a moderate risk of bias (moderate quality) and four or less as indicating a high risk (low quality) [19, 25]. Quality was assessed independently by two of the three authors (SvdW, AH, PK), and disagreements were resolved through discussion.

### Data synthesis

For each of the physical activity outcomes, the pre- and postoperative data regarding physical activity measurements, PROMs and percentage and timing of RTS and RTW were described. Studies described preoperative scores as the moment before surgery. When possible, the outcome of studies was pooled. Meta-analyses were performed using Review Manager 5.4 (RevMan, The Cochrane Collaboration 2020) by calculating the overall mean difference (MD) for the pre- and postoperative PROMs including a 95% confidence interval (CI), using a random effects model with the inverse variance approach. Missing mean scores and standard deviations for studies were imputed based on mean scores and standard deviations from other included studies with identical PROMs and study design. If a crosswalk between two or more PROMs was available, this was used to perform pooled analyses [32]. Additionally, PROMs were standardized by calculating the outcome with a minimum score of zero, meaning ‘the worst physical functioning’ and a maximum score of ten, meaning ‘the best physical functioning’, presuming that all included scales were linear. The included studies were divided into subgroups based on their study design and methodological quality.

### GRADE

The Grading of Recommendations Assessment, Development, and Evaluation (GRADE) framework was used to assess the quality of evidence and determine the strength of recommendations regarding the association between rKA and the level of physical activity. GRADE has four categories of certainty: high, moderate, low and very low [33].

The GRADE framework was drafted by one author (SvdW) and independently checked by a second author (PK) and any disagreements were resolved through discussion. The starting point for certainty was defined as ‘high’, corresponding with ‘We are very confident that the true effect lies close to that of the estimate of the effect’ [33], since all included studies aimed to identify the association between rKA and physical activity. Subsequently, the quality of evidence was downgraded based on five factors: 1. study limitations (majority of studies having a moderate or unclear risk of bias or minority of studies having a prospective study design), 2. inconsistency (I^2^ > 50%), 3. indirectness (population not fully representative of rKA patients or physical activity self-reported in the majority of patients), 4. imprecision (majority of studies having less than 100 revision operations or no precise estimate of effect size) and 5. publication bias is strongly suspected (yes). The quality of evidence was upgraded based on two factors: 1. moderate or large effect size (defined as an increase of 10% or more on an activity scale from 0–10) [34] and, 2. adjusted for confounders (majority of studies corrected for at least the three confounders age, BMI, and pre-operative activity level).

## Results

### Screening process

**Fig. 1:**
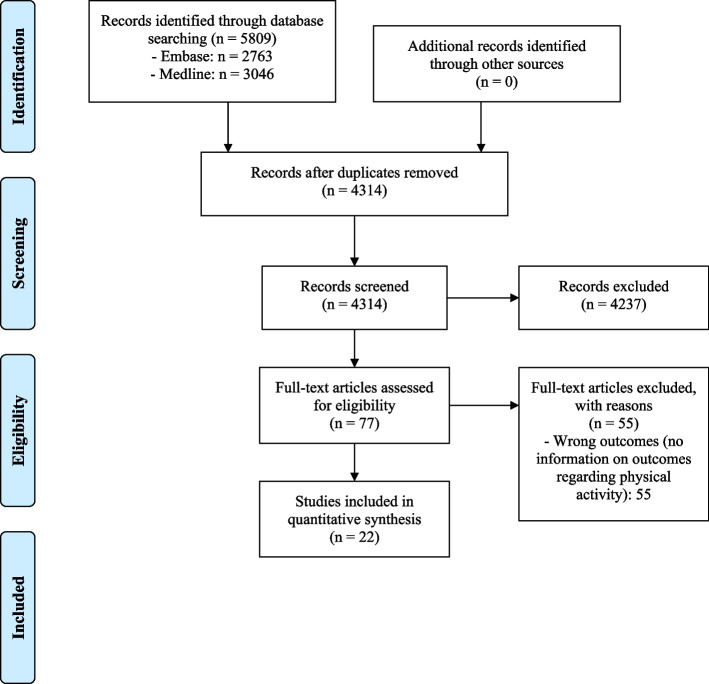
PRISMA Flow diagram

A total of 5,809 possibly relevant articles were identified with the primary search via Embase (2,763) and Medline (3,046). After removing 1,492 duplicates, the remaining 4,314 articles were screened, and the full text of 77 articles was assessed for eligibility. Finally, 22 articles were included (Fig. 1).

### Study characteristics

Of the 22 included studies, two studies were performed in Germany [35, 36], ten in the USA [7, 16, 37–44], four in Canada [45–48], two in the United Kingdom [49, 50], two in France [51, 52], one in Finland [53], and one in Russia [54] (Table 1). All studies were observational, of which 16 were retrospective cohort studies [16, 35–37, 40, 42, 43, 45–49, 52–54], and six were prospective cohort studies [7, 38, 39, 41, 44, 50]. The total number of patients in the 22 studies was 2,462 (range 14 – 308). The mean age of these patients was 67.2 years (range 24 – 95), and 1,425 women (58%) and 1,037 men (42%) were included. The mean follow-up of the studies was 3.5 years (range 0.5 – 9.1). Only patients who underwent rTKA were described and no studies reported on rUKA patients. None of the studies reported objective physical activity measurements, all studies reported PROMS, one study reported information on RTS [37], and two studies on RTW [37, 49].

**Table 1:** Pre- and postoperative outcomes: data extraction from the included studies

Study	Study design	Study population	Operation Type	Outcome Measures	Preoperative score	Postoperative score	Comparison	RTS + time to RTS	RTW + time to RTW	Confounding	Conclusion
Fuchs et al. [35] 2001; Germany [German]	Retrospective; follow-up: 8.5 m (range 6.5–61.4 m)	14 pts who underwent revision TKA Age: 67.4y (range, 54–78 years) Sex: 3 (21%) male 11 (79%) female	Revision TKA	HSS KSFS Tegner Activity Score Patella Score VAS	Unknown	67.5 (± 12.3) (*p* = 0.0002) * 57.0 (± 16.9) (*p* = 0.0002) * 1.3 (± 0.8) (*p* < 0.0001) * 19.1 (± 7.9) (*p* < 0.0001) * 5.8 (± 7.9) (*p* < 0.0001) *	Healthy volunteers 93.1 (± 10.3) 95.5 (± 15.1) 3.4 (± 0.8) 29.5 (± 1.5) 9.9 (± 0.3)	Unknown	Unknown		Functional deficits may be caused by preoperative deficits and are not only due to the operation
Barrack et al. [40] 2004; USA	Retrospective; follow up: 60 m (range, 24 – 84)	143 pts who underwent revision TKA Age: 69.7y Sex: 46 (32%) men, 97 (68%) women	Revision TKA with a revision of both the femoral and tibial components	**UCLA**		**5.3**		RTS unknown Time to RTS unknown	RTW unknown Time to RTW unknown		Stem design does appear to impact the incidence of end-of-stem pain in revision total knee arthroplasty
Dahm et al. [37] 2007; USA	Retrospective; follow-up: 5.6y (range, 3–9)	206 pts who underwent revision TKA between 1995 and 2000 Age: 69y (range, 31–87 years) Sex: 107 (52%) male, 99 (48%) female	Revision TKA	**UCLA** KSFS Patient Satisfaction	Unknown	**6.7 (range, 2–10)** (NS) * 62 (range, 0–100) (*p* < 0.001) * 77% (*p* < 0.001) *	Primary TKA 7.1 (range, 1–10) 71 (range, 0–100) 91%	RTS: Individual activity mentioned. Time to RTS not reported	RTW: 96% Time to RTW not reported	Mentioned, not adjusted for: patient selection, time since revision Adjusted for in analysis: age, BMI, comorbidities, sex	The average UCLA activity level rating was 6.7, which represents participation in active events such as swimming and bicycling
Mulhall et al. [38] 2007; USA	Prospective cohort study; follow-up: 6 m	186 pts who underwent revision TKA Age: 68.0y (range, 24.5–89.0y) Sex: 93 (50%) male, 93 (50%) female	Revision TKA	SF-36, PCS WOMAC Function KSFS **LEAS**	31.2 (± 7.3) 33.9 (± 14.2) 40.4 (± 21.4) **7.6 (± 2.5)**	37.2 (± 9.4) 21.9 (± 15.1) 62.7 (± 25.4) **8.5 (± 2.6)** **(*p* = 0.001)**		Unknown	Unknown		Improvement following rTKA is a multidimensional structure
Ghomrawi et al. [39] 2009; USA	Prospective cohort study; follow-up: 2y	308 pts who underwent revision TKA, 221pts with 2y follow-up Age: 68.7y (range, 34–85) Sex: 139 (45%) male, 169 (55%) female	Revision TKA	SF-36 PCS WOMAC Function **LEAS**	28.4 (± 7.5) 34.8 (± 13.8) **7.5 (± 2.6)**	34.8 (± 9.9) (*p* = 0.01) 23.8 (± 16.1) (*p* = 0.01) **8.7 (± 2.8)** **(*p* = 0.01)**		Unknown	Unknown	Mentioned, not adjusted for: surgeon experience Adjusted for in analysis: age, BMI, comorbidities, reason for revision, sex	The cohort of patients showed significant improvement in several functional outcomes in their first year following revision, however, the magnitude of the improvement was smaller than that after primary surgery
Gooding et al. [45] 2011; Canada	Retrospective; follow-up: 9y (range, 5-12y)	110 pts who underwent revision TKA, 48pts completed the outcome score questionnaires Age: 68y (range, 35-86y) Sex: 60 (55%) male, 50 (45%) female	Revision TKA	WOMAC Function OKS **UCLA** Patient Satisfaction	42.6 32.1	63.2 (*p* = 0.001) 60.8 (*p* = 0.0003) **4.1** 70.8		Unknown	Unknown	Mentioned not adjusted for: limited follow-up	Improvement was observed in the postoperative WOMAC function, pain, and global scores as well as the Oxford, SF-12 (mental) scores, and the satisfaction scores at last follow-up
Richards et al. [46] 2011; Canada	Retrospective; follow-up: 2y (range, 24-98 m)	24 pts who underwent revision TKA with FHSA Age: 72.8y Sex: 11 (46%) male, 13 (54%) female	Revision TKA with FHSA	WOMAC Function OKS SF-12 PCS Satisfaction function Satisfaction overall **UCLA**	Unknown	76 (*p* = 0.011) * 80 (*p* = 0.001) * 40 (*p* = 0.027) * 94 (*p* < 0.001) * 93 (*p* = 0.001) * **UCLA: 4.9** **(NS)** *	Revision TKA without FHSA 61 80 33 66 71 4.0	Unknown	Unknown	Mentioned, not adjusted for: lack of preoperative scores and difference in severity of preoperative bony defect	Patients with revision TKA with femoral head structural allograft had significant improved clinical outcomes compared to patients with revision TKA without femoral head structural allograft
Efe et al [36] 2012; Germany	Retrospective; follow up: 56 m ± 37 (range, 10 – 133)	28 pts who underwent revision TKA Age: 72.5y Sex: 33 (37%) men, 56 (63%) women	Revision TKA	**UCLA**		**4.1 ± 1 (range, 2 – 7)**		RTS unknown Time to RTS unknown	RTW unknown Time to RTW unknown		
Baker et al. [47] 2013; Canada	Retrospective; follow-up: 58 m (range, 24-123 m)	42 pts who underwent revision TKA with isolated polyethylene tibial insert exchange Age: 68y (range, 43-90y) Sex: 19 (45%) male, 23 (55%) female	Revision TKA with isolated polyethylene tibial insert exchange	OKS **UCLA** SF-12 PCS WOMAC Patient Satisfaction	45 (range, 13–75) **4 (range, 2–5)** 29 (range, 20–47) 50 (range, 9–84)	75 (range, 6–100) (*p* < 0.001) **6 (range, 1–10)** **(NS)** 39 (range, 18–58) (*p* = 0.007) 74 (range, 4–100) (*p* = 0.001) 86		Unknown	Unknown		When patients are selected appropriately, an isolated liner exchange can significantly improve the function of the knee
Hitt et al [41] 2014; USA	Prospective; follow up: 6 m	95 pts who underwent revision TKA Age: 67.5y (range, 49 – 92y) Sex: 44 (46%) men, 51 (54%) women	Revision TKA	**LEAS** KOOS Sport	**7.7 ± 2.6** 10.4 ± 18.4	**8.8 ± 2.4** **(*p* = 0.0027)** 36.8 ± 28.8 (*p* < 0.0001)		RTS unknown Time to RTS unknown	RTW unknown Time to RTW unknown		Patient expectations for improved outcomes are seen not only in primary replacement but also in revision cases
Stambough et al. [16] 2014; USA	Retrospective; follow-up: 4.6y (range, 2–13.4y)	76 pts (81 knees) who underwent revision TKA Age: 48.5y (range, 25-55y) Sex: 28 (35%) male, 53 (65%) female	Revision TKA	KSFS **UCLA**	46 (range, 5–100) (NS) **3.3 (range, 1–9)** **(NS)**	50.7 (range, 5–100) (*p* < 0.001) **4.1 (range, 1–10)** **(NS)**	Primary TKA pre-op: 45.6 (range, 20–80) post-op: 66.6 (range, 20–100) pre-op: 3.5 (range, 1–9) post-op: 4.7 (range, 2–10)	Unknown	Unknown	Mentioned, not adjusted for: Different surgical techniques and designs of implant	Young patients undergoing revision TKR should be counselled that they can expect somewhat less improvement and a higher risk of complications than occur after primary TKR
Grayson et al [42] 2016; USA	Retrospective; follow up: 21 m (range, 9 – 54)	92 pts who underwent revision TKA Age: 65.2y (range, 40 – 89y) Sex: 33 (37%) men, 56 (63%) women	Revision TKA	**UCLA**	**2.7**	**4.7**		RTS unknown Time to RTS unknown	RTW unknown Time to RTW unknown	Adjusted for in analysis: reason for revision	Overall, our findings indicate that patients with flexion instability functionally improve after revision TKA but that they do not appear to improve as much relative to their higher baseline function as patients revised for infection or loosening/osteolysis
Sandiford et al. [48] 2017; Canada	Retrospective; follow-up: 9y (range, 5-12y)	30 pts who underwent revision TKA with FHSA Age: 66y (range, 30-85y) Sex:	Revision TKA with FHSA	OKS WOMAC **UCLA** Patient Satisfaction	Unknown	80 (range, 69–100) (NS) * 82.3 (range, 75–100) (NS) * **5.8 (range, 3–8)** (NS) * 93 (range, 33–100) (NS)	Revision TKA with trabecular metal cone 84 (range, 56–100) 84.6 (range, 58–100) 5.5 (range, 3–8) 95.2 (range, 66–100)	Unknown	Unknown	Mentioned, not adjusted for: patient selection, time since revision Adjusted for in analysis: reason for revision	No difference was found in pain, function, or repeat revision when comparing femoral head allografts and trabecular metal cones for severe bone defects during revision TKA
Scott et al. [49] 2018; United Kingdom	Retrospective; follow-up: 3.8y (range, 1-9y)	30 pts who underwent revision TKA Age: 58y (range, 46-64y) Sex: 16 (53%) male, 14 (47%) female	Revision TKA	OKS **UCLA**	17.1 (range, 4–33) **4.3 (range, 1–10)**	OKS: 23.4 (range, 5–47) **5.1 (range, 1–10)**		Unknown	RTW 2/28 (7.1%) after one year. RTW 5/28 (18%) after 3.8 year Time to RTW: 2 within 1 year, 3 after 1 year	Mentioned, not adjusted for: preoperative intentions regarding returning to work Adjusted for in analysis: age, sex	Very few patients RTW after revision TKA
Turnbull et al. [50] 2019; United Kingdom	Prospective; follow-up: 3.9y (range, 1.2–11.8y)	112 pts who underwent revision TKA Age: 71y (range, 47-94y) Sex: 67 (56%) male, 45 (44%) female	Revision TKA	OKS **UCLA**	OKS: 15 (range, 0–46) **UCLA: 4.4 (range, 2–6)**	OKS: 27 (range, 5–42) (*p* < 0.001) **UCLA: 5.3 (range, 3–6)** **(*p* < 0.001)**		Unknown	Unknown	Adjusted for in analysis: age, BMI, sex, number of comorbidities, reason for revision, number of comorbidities,	Although 90% of the patients maintain activity levels following revision TKA, less than half increase levels and this is predicted by male sex and pre-revision activity levels
Jacquet et al. [51] 2020; France	Retrospective; follow-up: 9.1y ± 2.1	99 pts who underwent revision TKA Age: 72y ± 2 Sex: Unknown	Revision TKA	KOOS Sport	13.9 ± 1.4	35.6 ± 2.6		RTS unknown Time to RTS unknown	RTW unknown Time to RTW unknown		
Piuzzi et al. [7] 2020; USA	Prospective; follow-up: 1y	246 pts who underwent aseptic revision TKA Age: 64.9y (range, 55-74y) Sex: 105 (42.7%) male, 141 (57.3%) female	Revision TKA	KOOS Pain KOOS PS KOOS-QOL VR-12 PCS VR-12 MCS	39.9 (± 19.9) 45.9 (± 17.8) 18.5 (± 18.8) 26.7 (± 8.28) 45.8 (± 13.2)	70.2 (*p* < 0.001) 65.1 (*p* = 0.007) 48.2 (*p* = 0.024) 36.07 (*p* = 0.012) 50.2 (*P* = 0.033)		Unknown	Unknown		Although overall QOL improved, other global-health PROMs remained unchanged
Erivan et al. [54] 2021; Russia	Retrospective; follow-up: ≥ 2y	61 pts who underwent revision TKA Age: 60.4y (range, 34 – 80y) Sex: 30 (49%) men, 31 (51%) women	Revision TKA with a revision of the tibial component	Devane KOOS	56.3 ± 10.8 (range, 36.9 – 66)	2.6 71.8 ± 22.6 (range, 24.9 – 100) (*p* = 0.013)		RTS unknown Time to RTS unknown	RTW unknown Time to RTW unknown		The present study of cones used for tibial revision supports shows excellent results; however, longer and larger follow-up is needed to better assess results in revision TKA
Houfani et al. [52] 2021; France	Retrospective; follow-up: 67.3 m ± 11.8 (range, 13–180)	127 pts who underwent revision TKA Age: 69.5y (range, 42 – 89y) Sex: 48 (38%) men, 79 (62%) women	Revision TKA	Devane	2.7 ± 1 (range, 1 – 5)	2.26 (*p* = 0.0003)		RTS unknown Time to RTS unknown	RTW unknown Time to RTW unknown		
Sonn et al. [43] 2021; USA	Retrospective; follow-up: 18.5 m (range, 11.0 – 78.7)	107 pts who underwent revision TKA, with ≥ 50% pain relief with injections Age: 62.6y (range, 35 – 92y) Sex: 52 (36%) men, 92 (64%) women	Revision TKA	**UCLA** KOOS Jr	**3.5** **(NS)** 40.7 (NS)	**4.8** **(NS)** 60.9 (NS)	37 pts who underwent revision TKA, with ≤ 50% pain relief with injections **UCLA pre-op** **4.0** **(NS)** **UCLA post-op** **4.3** **(NS)** KOOS Jr pre-op 44.4 (NS) KOOS Jr post-op 56.6 (NS)	RTS unknown Time to RTS unknown	RTW unknown Time to RTW unknown	Adjusted for in analysis: reason for revision, prevalence of comorbidities	Study results showed that patients reporting > 50% pain relief after a diagnostic injection have significantly better improvement in UCLA Activity Level at minimum 1- year follow-up
Von Hitze et al. [53] 2021; Finland	Retrospective; follow-up: 7.3y (range, 4 – 12.7)	119 pts who underwent revision TKA Age: 71.7y (range, 31 – 95y) Sex: 33 (28%) men, 86 (72%) women	Revision TKA, using the single rotating hinged knee design	KOOS Sport		33 (range, 0 – 100)		RTS unknown Time to RTS unknown	RTW unknown Time to RTW unknown		
Auran et al. [44] 2022; USA	Prospective; follow-up: 2y	181 pts who underwent revision TKA Age: 66y (range, 45 – 92y) Sex: 70 (39%) men, 111 (61%) women	Revision TKA	**LEAS**	**7.8**	**9.5**		RTS unknown Time to RTS unknown	RTW unknown Time to RTW unknown		

Values in bold were included in the meta-analysis

*BMI* Body Mass Index, *FHSA* Femoral head structural allograft *HSS* Hospital for Special Surgery *KA* Knee arthroplasty *KOOS* Knee Injury and Osteoarthritis Outcome Score *KOOS PS* Koos Physical Function Shortform *KSFS* Knee Society Function Score *LEAS* Lower Extremity Activity Scale *MCS* Mental component score *NS* Not significant *OKS* Oxford Knee Score *PCS* Physical component score *PKA* Primary knee arthroplasty *PROMs* Patient reported outcome measures *QOL* Quality of life *RT*S Return to sports *RTW* Return to work *SF-12* twelve item short form survey *SF-36* thirty-six item short form survey *TKA* Total knee arthroplasty *TKR* Total knee replacement *UCLA* University of California at Los Angeles *UKA* Unicondylar knee arthroplasty *VAS* Visual Analogue Scale *VR-12 MCS* Veterans RAND-12 mental component score *VR-12 PCS* Veterans RAND-12 physical component score *WOMAC* Western Ontario and McMaster Universities Osteoarthritis Index *rTKA* Revision total knee arthroplasty

^*^p-value based on comparison of study population and ‘comparison’

### Quality assessment

None of the included studies had a low risk of bias, 20 studies had a moderate risk [7, 16, 36–44, 46–54] and two studies had a high risk [35, 45]. Most stars (equivalent to the lowest risk of bias) were awarded for the item “ascertainment of exposure”, since all 22 studies reported on rKA patients. The least number of stars (equivalent to highest risk of bias) was awarded for the item “assessment of outcome”, since no study reported objective physical activity measurements, and all studies described self-reported physical activity data (Additional file 2).

The total number of patients in the 20 moderate quality studies was 2,338 (range 24 – 308). The mean age of the patients was 67.2 years (range 24 – 95), including 1,364 women (58%) and 974 men (42%). The mean follow-up of the studies was 3.3 years (range 0.5 – 9.1).

The total number of patients in the two low quality studies was 124 (range 14 – 110). The mean age of the patients was 67.9 (range 35 – 86), including 61 women (49%) and 63 men (51%). The mean follow-up of the studies was 8.1 years (range 0.7 – 9) (Table 1). The range of follow-up times reflects the range of the mean follow-up time per study, and not that of the individual patient within each study.

### Physical activity

Most studies reported on the UCLA score (12 studies) [16, 36, 37, 40, 42, 43, 45–50], four studies reported on LEAS [38, 39, 41, 44], four studies reported on activity-related KOOS subscales [7, 41, 43, 51, 53, 54], two studies reported on the Devane activity score [52, 54], and one study reported on the Tegner activity score [35]. The 12 studies describing UCLA scores (*n* = 965) found a mean UCLA score of 3.3 (± 1.9) preoperatively and 5.2 (± 2.3) postoperatively. The LEAS score was described in four studies (*n* = 669), with a preoperative mean of 7.6 (± 2.6), and a postoperative mean of 8.9 (± 2.7).

The KOOS-Sport was reported in three studies [41, 51, 53] (*n* = 313), with a preoperative mean of 12.2 (± 13.0), and postoperative mean of 35.0 (± 20.0). The KOOS-PS was reported by Piuzzi et al. [7] (*n* = 313), with mean preoperative and postoperative scores of 45.9 (± 17.8) and 65.1 (± 22.5) respectively.

The Devane activity score was used in two studies [52, 54] (*n* = 188), with a mean of 2.7 (± 1.0) preoperatively and a mean of 2.4 postoperatively. The Tegner activity score was described once (*n* = 14). Fuchs et al. only reported a postoperative Tegner activity score of 1.3 (± 0.8) [35].

**Fig. 2:**
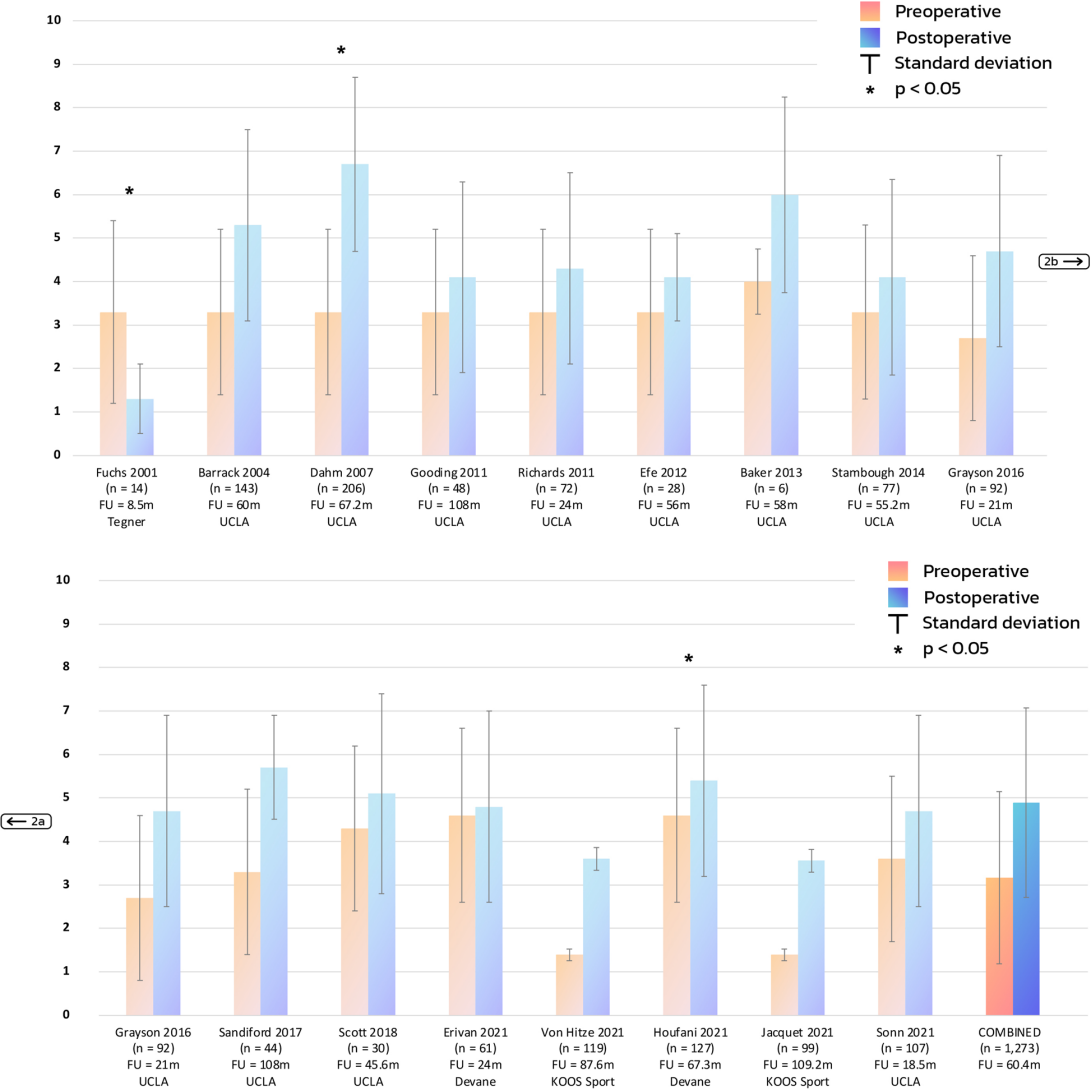
Physical activity: mean and standard deviation for included retrospective studies and their respective outcome measure. *Devane* Devane activity score *FU* follow-up *KOOS Sport* Knee Injury and Osteoarthritis Outcome Score Function in Sport *M* months *N* number *Tegner* Tegner activity score *UCLA* University of California at Los Angeles activity scale

**Fig. 3:**
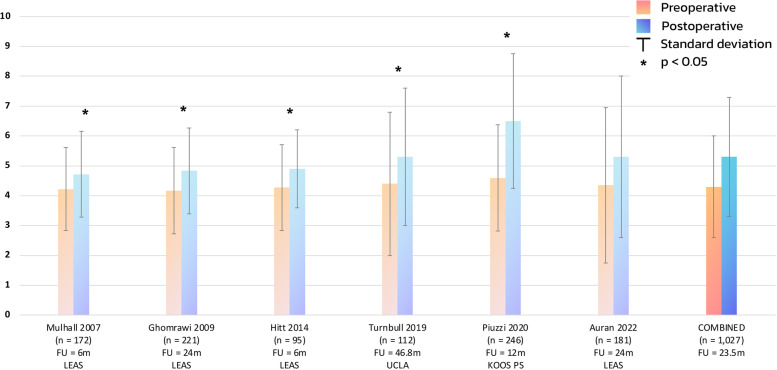
Physical activity: mean and standard deviation for included prospective studies and their respective outcome measure. *FU* follow-up *KOOS PS* Knee Injury and Osteoarthritis Outcome Score Physical Function Shortform *LEAS* Lower-Extremity Activity Scale *M* months *N* number *UCLA* University of California at Los Angeles activity scale

The combined total of the 16 retrospective studies resulted in a mean of 3.2 (± 2.0) preoperatively and a mean of 4.9 (± 2.2) postoperatively (Fig. 2). Moreover, the combined total of the two low-quality retrospective studies resulted in a mean of 3.3 (± 1.9) preoperatively and a mean of 3.5 (± 2.2) postoperatively. Additionally, the combined total of the 14 retrospective studies of moderate-quality resulted in a mean of 3.2 (± 2.0) preoperatively, and a mean of 5.0 (± 2.2) postoperatively. The combined total of the six prospective studies resulted in a mean of 4.3 (± 1.7) preoperatively, and a mean of 5.3 (± 2.0) postoperatively (Fig. 3).

**Fig. 4:**
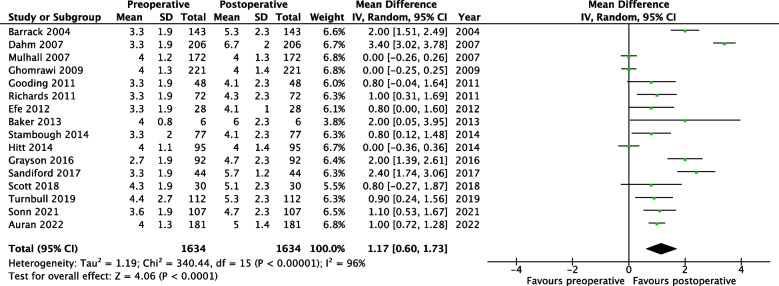
Forest plot of meta-analysis: physical outcome measures for studies describing LEAS or UCLA activity scores. *CI* Confidence Interval *IV* Inverse variance *SD* Standard deviation

**Fig. 5:**
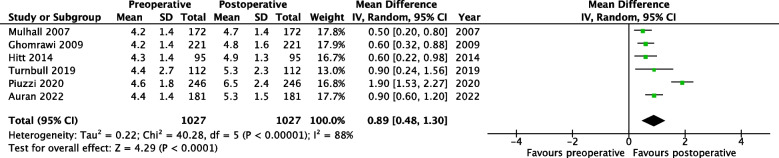
Forest plot of meta-analysis: physical outcome measures for prospective studies. *CI* Confidence Interval *IV* Inverse variance *SD* Standard deviation

Figures 4 and 5 present the meta-analyses performed for the subgroups. A crosswalk was used to derive UCLA scores from LEAS scores of four prospective studies [32]. The derived scores were used in a meta-analysis, combined with UCLA scores described by one prospective study and 11 retrospective studies. The crosswalk subgroup (Fig. 4) included a total of 16 studies and showed a significant postoperative increase of 1.2 points (MD = 1.17, 95% CI 0.60 – 1.73 (*p* < 0.0001)).

Furthermore, the prospective subgroup included six studies and showed a significant postoperative increase of 0.9 points (MD = 0.89, 95% CI 0.48 – 1.30 (*p* < 0.0001)), from ‘regular participation in mild activities such as walking, limited housework, and limited shopping’ to ‘sometimes participates in moderate activities’ (Fig. 5).

### Return to sport

One study reported on RTS. Dahm et al. [37] reported individual athletic activities for 206 rTKA patients. No preoperative data on activity-specific participation were reported. Out of the 206 patients, 87% reported participating in slow walking after rTKA, while 54% of the patients participated in medium paced walking. Additionally, 12% of patients engaged in non-recommended activities (i.e., hockey, soccer, football, gymnastics, jogging, singles tennis, and basketball) after rTKA. The time to RTS was not mentioned in the study.

### Return to work

Two studies reported on RTW. The pooled mean RTW of the two studies was 86%. Dahm et al. [37] reported a RTW percentage of 96% after 5.6 years (range 3 – 9), and 67% of rTKA patients had a work level comparable to activities of daily living. Moreover, Dahm et al. [37] found that 9% of the patients participated in heavy manual labour after revision and 24% participated in light manual labour after revision. Scott et al. [49] reported an RTW percentage of 18%, with an RTW rate of 7% one year after rTKA. In addition, no statistically significant reasons for not returning to work were reported. None of the patients returned to heavy manual labour after revision.

### Confounding factors

Of the 22 included studies, nine adjusted for possible confounding factors that could affect physical activity (Table 1). These confounders included age, sex, BMI, (number of) comorbidities, preoperative activity, the reason for revision and time since revision. Three of the 22 studies identified age as a possible confounding factor. Dahm et al. [37] controlled for age as a confounding factor but did not find a significant difference between patients > 70 years and patients < 70 years. Turnbull et al. [50] reported that age did not affect UCLA activity scores, however, younger patients (< 65 years) were less likely to be satisfied with physical activity after revision. Ghomrawi et al. [39] found that age did not influence LEAS scores. Four of the included studies reported sex as a possible confounder. Male patients had higher average postoperative UCLA scores than female patients [37, 39, 49, 50]. Three studies identified BMI as a potential confounding factor, although no correlation was found between BMI and physical activity in these studies [37, 39, 50]. The prevalence of comorbidities was described as a possible confounder in three studies. Dahm et al. [37] reported that 61% of the included patients were limited in physical activity due to other joints. Ghomrawi et al. [39] reported that patients with a higher number of comorbidities were less likely to be physically active after revision. Sonn et al. stated that rTKA patients with > 50% pain relief after injections, self-reported improvement in activity level and maintained greater satisfaction after a minimum of one year, when compared to rTKA patients with < 50% pain relief after injections [43]. Preoperative scores regarding physical activity were analysed in 16 studies [7, 16, 38, 39, 41–44, 47, 49–52, 54]. Five studies mentioned the reason for revision as a possible confounding factor. Ghomrawi et al. [39] reported that patients with a failed KA due to malalignment, had higher average postoperative LEAS scores. Sandiford et al. [48] mentioned that patients with trabecular metal cones could have better outcomes, since this type of KA was used for more simple defects. Turnbull et al. [50] stated that UCLA activity levels were not affected by the reason for revision. Grayson et al. reported that the reason for revision was not significant for preoperative and postoperative differences [42]. Sonn et al. mentioned that instability cases showed a significantly higher improvement in UCLA activity level when compared to aseptic loosening cases, from preoperative to a minimum follow-up of one year [43].

Time since revision was mentioned as a possible confounder in two studies. Dahm et al. [37] described that patients had undergone a revision in the previous three to ten years, and mentioned that the percentage of good results continued to increase up until 60 months following revision. Sandiford et al. [48] reported that the five year follow-up may not have been long enough to include all types of failure occurring in patients with revision.

### GRADE

The quality of evidence in the sixteen studies using the LEAS and UCLA crosswalk was rated as very low according to the GRADE framework, due to four downgrades and one upgrade (Additional file 3). This is the equivalent of ‘We have very little confidence in the effect estimate: the true effect is likely to be substantially different from the estimate of effect’ [33]. For the six prospective studies, the quality of evidence was rated as low, with two downgrades and no upgrades (Additional file 3). This is the equivalent of ‘Our confidence in the effect estimate is limited: the true effect may be substantially different from the estimate of the effect’ [33].

## Discussion

The most important finding of the present study was that physical activity following rTKA was equal or higher compared to preoperative physical activity, as evaluated by the pooled PROM analyses. Limited data suggest that patients can also return to low impact sports and work activities after rTKA. No studies in this systematic review included UKA patients, therefore no comparisons could be made between rUKA and rTKA.

### Physical activity

This study showed an improvement from pre- to postoperatively self-reported physical activity after rTKA. Although both pTKA and rTKA generally lead to improvement in function, two previous studies reported that the extent of improvement regarding physical function is inferior after rTKA compared to pTKA [20, 55]. The first study found an overall 12% lower score for the revision group using WOMAC, Oxford Knee and SF-12 scores. The second study found a worse pain score in the revision group, but a similar American Knee Society Score and SF-12 score [54]. We did not include these overall function scores in the present study given our focus on physical activity. Therefore, we cannot compare the physical activity scores as presented in our review with these overall function scores. However, given the uncertainty of our findings based on GRADE, these less favourable outcomes when comparing rTKA versus pTKA are important to consider when discussing the option of pKA, especially for patients with a higher risk of rKA.

Konings et al. reported a pooled mean UCLA score of 6.5 postoperatively (± 2.1) for patients with a pKA, equivalent to regular participation in active events such as bicycling [56]. Our review included studies with mean UCLA activity scores postoperatively ranging from 4.1 to 6.7, which is equivalent to ‘regular participation in mild activities, such as walking, limited housework, and limited shopping’ and ‘regular participation in active events, such as bicycling’ respectively. This review showed a mean of 5.2 (± 2.3) for postoperative UCLA activity scores, equivalent to scores from mild activity to regular participation in active events such as bicycling.

Based on limited available data, mean UCLA scores after rTKA appeared to be comparable to UCLA scores after pTKA, suggesting comparable levels of postoperative physical activity. This is an encouraging result, although larger studies, preferably with activity monitors, should confirm our present findings. Twiggs et al. described this type of physical activity measurement for patients with pTKA [57], and a similar approach could be used for patients with rKA.

### Return to sport and work

One study reported on RTS, and two studies reported on RTW for rTKA patients. This limited amount of research is noteworthy since an increasing number of younger patients will undergo rTKA. Dahm et al. reported that 12% of the enrolled patients participated in sports, which were categorized as ‘not recommended’ [37]. Also, self-assessment of activity versus peers showed that patients reported a slightly higher activity level after rTKA compared to their age group [37]. This indicates that, after rTKA, patients estimated themselves to be at least as active as their respective age group. After KA, high impact activities are generally discouraged due to a higher risk of revision [58]. However, conclusive evidence on the influence of sports on the lifespan of knee implants is lacking. A recent study described higher implant survivorship for highly active TKA patients compared to patients with low activity following TKA [59]. This shows that limiting physical activity may not be necessary for patients with modern-day KA implants. Nonetheless, caution is advised for patients with rTKA who want to pursue high-impact activities, as this might increase the chance of requiring re-revision.

Two studies reported data on RTW and the pooled mean RTW percentage of this review was 86%. Scott et al. reported a very low RTW rate (7%) but ﻿mentioned that 71% of the patients had retired and 21% were on welfare benefits after one year.

Due to the limited information available on RTW, no distinction can be made between RTW for pTKA and rTKA patients. To provide more reliable estimates for RTS and RTW after rTKA, more studies are needed.

Patients should be well informed and guided following rTKA. Rehabilitation programmes might contribute to a better outcome regarding RTS and RTW. However, the degree to which these programmes contribute to RTS and RTW for rTKA is unknown. Even for pTKA, no research was found on the effect on RTS and RTW [60]. To prevent unmet expectations and improve patient satisfaction following rTKA or rUKA, setting patient specific goals prior to revision could be beneficial [61]. Making use of ‘goal attainment scaling (GAS)’ during rehabilitation, for example, resulted in higher patient satisfaction with work-activities compared with standard rehabilitation [62–64].

High-impact activities are generally discouraged due to a higher risk of revision [58]**,** although a recent review disputes this [65]**.** Dahm et al. stated that high-impact activities following rTKA are possibly even more concerning [37]. However, evidence on the influence of leisure time and occupational physical activity on the lifespan of rTKA is lacking. Therefore, caution is advised for patients with rTKA who want to pursue high impact activities in leisure time and work, as this could result in an increased chance for re-revision. Due to the limited lifespan of the implant and the possible increased risk of reoperation, revision, and re-revision for younger patients, non-operative treatments should be considered to postpone pKA and rKA [18]. Currently, non-surgical treatment before KA remains underutilized, although this could contribute to a higher participation in sports and work, and delay pKA and rKA [66]. Furthermore, other techniques than KA could be considered when treating younger patients with knee OA. A recent study by Hoorntje et al. showed positive results of both osteotomies and knee joint distraction as possible joint-preserving options for young end-stage knee OA patients [67].

### Strengths and limitations

This study presents the first meta-analysis of data on physical activity after rTKA. Furthermore, the included studies and their respective scores were divided into subgroups based on methodological quality. An important limitation of the present study is the risk of bias in the included studies and the uncertainty of the outcomes found according to GRADE. None of the included studies were of high methodological quality. Most of the included studies were of moderate or low quality, and the GRADE score was very low and low. All outcome measures presented in the included studies were patient-reported, which increases the risk of recall bias. Due to this risk, PROM activity scores may have been overestimated or underestimated by the patients. Unfortunately, no objective physical activity measurements were performed in the included studies. Therefore, significant associations between activity scores and change in activity could be unrightfully assumed. Additionally, a limitation is the missing preoperative scores of nine of the 22 included studies. Therefore, missing preoperative scores and standard deviations were based on scores and standard deviations of included studies with a similar study design and outcome measures among similar patients. Furthermore, pooling various outcome measures to assess physical activity may contribute to a less reliable outcome of this study. However, previous studies similarly assessed physical activity using normalised scales [68]. Another limitation of this review is the inclusion of studies with various types of implants. These varying implants may not be directly comparable, which needs to be considered when interpreting our findings. An additional limitation of this study is that only two databases were used, namely Medline and Embase. By not considering grey literature and pre-print repositories, we might have overlooked studies that could have been included in this review. Furthermore, this study was not prospectively registered in the repositories, and the unpublished study protocol represents a limitation of this study. Additionally, no assessment tool was used to calculate the inter-rater reliability of the independent selection of potential eligible papers by the two authors. Finally, a limitation is the heterogeneity of the included studies, which resulted in a less reliable meta-analysis.

## Conclusion

This systematic review and meta-analysis showed that the majority of patients reported an improved activity level after rTKA and were able to maintain an active lifestyle in daily life, including sports and work. However, the substantial uncertainty, as rated via GRADE, should be considered when using these findings. To provide more reliable estimates for physical activity, RTS and RTW after rTKA, more prospective studies are needed that use objective physical activity measurements for both leisure time and occupational physical activity, given the expected strong rise in the number of younger and more demanding rTKA patients around the world.

## Supplementary Information

**Additional file 1. **Full description of performed search in Medline and Embase.

**Additional file 2. **Newcastle-Ottawa Quality Assessment Scale.

**Additional file 3. **GRADE results.

**Additional file 4. **Forest plot of meta-analysis: physical outcome measures for retrospective studies.

**Additional file 5. **Studies and reasons for exclusion following full text screening.

## Data Availability

The datasets supporting the conclusions of this article are included within this article and its additional files.

## Abbreviations

BMI: Body Mass Index
CI: Confidence interval
EQ-5D: EuroQol-5D
FHSA: Femoral head structural allograft
GRADE: Grading of Recommendations Assessment, Development and Evaluation
HSS: Hospital for Special Surgery
KA: Knee arthroplasty
KOOS: Knee Injury and Osteoarthritis Outcome Score
KOOS PS: Koos Physical Function Shortform
KSFS: Knee Society Function Score
LEAS: Lower Extremity Activity Scale
MCS: Mental component score
MD: Mean difference
NOS: Newcastle–Ottawa Scale
OA: Osteoarthritis
OKS: Oxford Knee Score
PCS: Physical component score
pKA: Primary knee arthroplasty
pTKA: Primary total knee arthroplasty
pUKA: Primary unicondylar knee arthroplasty
QOL: Quality of life
rKA: Revision knee arthroplasty
rTKA: Revision total knee arthroplasty
rUKA: Revision unicondylar knee arthroplasty
RTS: Return to sports
RTW: Return to work
RevMan: Review Manager
SF-12: Twelve item short form survey
SF-36: Thirty-six item short form survey
TKA: Total knee arthroplasty
TKR: Total knee replacement
UCLA: University of California at Los Angeles
UKA: Unicondylar knee arthroplasty
VAS: Visual Analogue Scale
VR-12 MCS: Veterans RAND-12 mental component score
VR-12 PCS: Veterans RAND-12 physical component score
WOMAC: Western Ontario and McMaster Universities Osteoarthritis Index
WORQ: Work, Osteoarthritis and Joint-Replacement Questionnaires
