# Supplementary material for: Physical activity after revision knee arthroplasty including return to sport and work: a systematic review and meta-analysis including GRADE
Source: BMC Musculoskelet Disord. 2023 May 9;24:368. doi: 10.1186/s12891-023-06458-y (PMC10170708; doi:10.1186/s12891-023-06458-y)
Supplement: Supplementary file 1 — Additional file 1. Full description of performed search in Medline and Embase. [file 12891_2023_6458_MOESM1_ESM.docx]

***Search strategy Medline:***

(("Arthroplasty, Replacement, Knee"[MeSH] OR "Knee Prosthesis"[MeSH] OR knee arthroplast*[tiab] OR knee replacement*[tiab] OR knee prosthes*[tiab] OR TKA[tiab] OR TKR[tiab] OR total knee[tiab]) AND ("Surgical Procedures, Operative"[Mesh:NoExp] OR "surgery" [Subheading] OR "Reoperation"[Mesh] OR reoperat*[tiab] OR revision*[tiab]) AND ("Recovery of Function"[Mesh] OR "Return to Sport"[Mesh] OR "Return to Work"[Mesh] OR "Prosthesis Failure"[Mesh] OR (return*[tiab] AND (sport*[tiab] OR work*[tiab])) OR recover*[tiab] OR activity level[tiab] OR patient outcome[tiab])) AND ("Severity of Illness Index"[Mesh] OR score*[tiab] OR scoring[tiab] OR assess*[tiab] OR rate*[tiab] OR rating[tiab] OR scale*[tiab]) AND (("Follow-Up Studies"[Mesh] OR "Cohort Studies"[Mesh] OR "Case-Control Studies"[Mesh] OR "Controlled Clinical Trial" [Publication Type] OR "Comparative Study" [Publication Type] OR "Observational Study" [Publication Type] OR "Systematic Review"[pt] OR follow up[tiab] OR followup[tiab] OR cohort[tiab] OR case control[tiab] OR case stud*[tiab] OR random*[tiab] OR controlled clinical trial*[tiab] OR controlled trial*[tiab] OR retrospective*[tiab] OR prospective*[tiab] OR observational stud*[tiab]))

***n = 3,046***

***Search Embase:***

| **#** | **Searches** | **Results** |
| --- | --- | --- |
| **1** | knee replacement/ or total knee arthroplasty/ or exp knee prosthesis/ or (knee arthroplast* or knee replacement* or knee prosthes* or TKA or TKR or total knee).ti,ab,kw. | 57129 |
| **2** | *surgery/ or su.fs. or reoperation/ or exp revision arthroplasty/ or (reoperat* or revision*).ti,ab,kw. | 2503752 |
| **3** | return to work/ or return to sport/ or convalescence/ or exp prosthesis complication/ or (recover* or activity level or patient outcome).ti,ab,kw. | 1063482 |
| **4** | (return* adj3 (sport* or work*)).ti,ab,kw. | 23265 |
| **5** | 3 or 4 | 1076595 |
| **6** | musculoskeletal disease assessment/ or oxford knee score/ or scoring system/ or (score* or scoring or assess* or rate* or rating or scale*).ti,ab,kw | 9102915 |
| **7** | exp controlled clinical trial/ or cohort analysis/ or exp case control study/ or follow up/ or observational study/ or comparative study/ or exp priority journal/ or major clinical study/ or (followup or follow up or random* or cohort or case control or case stud* or random* or controlled clinical trial* or controlled trial* or retrospective* or prospective* or observational stud*).ti,ab,kw. | 16485522 |
| **8** | 1 and 2 and 5 and 6 and 7 | 2763 |

***n = 2,763***
