# Supplementary material for: Physical activity after revision knee arthroplasty including return to sport and work: a systematic review and meta-analysis including GRADE
Source: BMC Musculoskelet Disord. 2023 May 9;24:368. doi: 10.1186/s12891-023-06458-y (PMC10170708; doi:10.1186/s12891-023-06458-y)
Supplement: Supplementary file 2 — Additional file 2. Newcastle-Ottawa Quality Assessment Scale. [file 12891_2023_6458_MOESM2_ESM.docx]

| **COHORT STUDIES** | | | |  |  |  |  | |  |  | |  |
| --- | --- | --- | --- | --- | --- | --- | --- | --- | --- | --- | --- | --- |
|  | **Selection** |  |  |  | **Comparability** | **Outcome** |  |  | | | **Total** | |
|  | *Representativeness* | *Selection of non-exposed cohort* | *Ascertainment of exposure* | *Outcome of interest not present at start of study* | *Comparability* | *Assessment* | *Follow-up length* | | *Adequacy of follow-up* | |  | |
|  |  |  |  |  |  |  |  | |  | |  | |
| Fuchs / 2001 | - | - | * | * | - | - | - | | - | | 2 | |
| Barrack / 2004 | * | - | * | * | * | - | * | | * | | 6 | |
| Dahm / 2007 | * | * | * | * | * | - | * | | * | | 7 | |
| Mulhall / 2007 | * | - | * | * | * | - | - | | * | | 5 | |
| Ghomrawi / 2009 | * | - | * | * | ** | - | * | | - | | 6 | |
| Gooding / 2011 | * | - | * | * | - | - | * | | - | | 4 | |
| Richards / 2011 | * | * | * | * | - | - | * | | * | | 6 | |
| Efe / 2012 | * | * | * | * | - | - | * | | * | | 6 | |
| Baker / 2013 | * | - | * | * | * | - | * | | * | | 6 | |
| Hitt / 2014 | * | * | * | * | ** | - | - | | * | | 7 | |
| Grayson / 2016 | * | - | * | * | ** | - | * | | * | | 7 | |
| Sandiford / 2017 | * | * | * | * | - | - | * | | * | | 6 | |
| Scott / 2018 | * | - | * | * | ** | - | * | | * | | 7 | |
| Turnbull / 2019 | * | - | * | * | ** | - | * | | * | | 7 | |
|  |  |  |  |  |  |  |  | |  | |  | |

|  | **Selection** |  |  |  | **Comparability** | **Outcome** |  |  | | **Total** |
| --- | --- | --- | --- | --- | --- | --- | --- | --- | --- | --- |
|  | *Representativeness* | *Selection of non-exposed cohort* | *Ascertainment of exposure* | *Outcome of interest not present at start of study* | *Comparability* | *Assessment* | *Follow-up length* | | *Adequacy of follow-up* |  |
|  |  |  |  |  |  |  |  | |  |  |
| Jacquet / 2020 | * | - | * | * | ** | - | * | | * | 7 |
| Piuzzi 2020 | * | - | * | * | ** | - | * | | - | 6 |
| Erivan / 2021 | * | - | * | * | * | - | * | | * | 6 |
| Houfani / 2021 | * | - | * | * | ** | - | * | | * | 6 |
| Sonn / 2021 | * | - | * | * | ** | - | * | | - | 6 |
| Von Hitze / 2021 | * | - | * | * | * | - | * | | - | 5 |
| Auran / 2022 | * | - | * | * | ** | - | * | | * | 7 |
|  |  |  |  |  |  |  |  | |  |  |

| **CASE-CONTROL STUDIES** | | | |  |  |  |  |  |  | |  |
| --- | --- | --- | --- | --- | --- | --- | --- | --- | --- | --- | --- |
|  | **Selection** |  |  |  | **Comparability** | **Exposure** |  |  | | **Total** | |
|  | *Case definition* | *Representativeness* | *Selection of controls* | *Definition of controls* | *Comparability* | *Ascertainment of exposure* | *Same method of ascertainment for cases and controls* | *Non-response rate* | |  | |
|  |  |  |  |  |  |  |  |  | |  | |
| Stambough / 2014 | * | * | - | * | * | * | * | - | | 6 | |
|  |  |  |  |  |  |  |  |  | |  | |
