# Supplementary material for: Physical activity after revision knee arthroplasty including return to sport and work: a systematic review and meta-analysis including GRADE
Source: BMC Musculoskelet Disord. 2023 May 9;24:368. doi: 10.1186/s12891-023-06458-y (PMC10170708; doi:10.1186/s12891-023-06458-y)
Supplement: Supplementary file 3 — Additional file 3. GRADE results. [file 12891_2023_6458_MOESM3_ESM.docx]

|  | | **Possible downgrades** |  |  |  |  |  |  |  | **Possible upgrades** |  | **Overall quality** |
| --- | --- | --- | --- | --- | --- | --- | --- | --- | --- | --- | --- | --- |
|  | |  |  |  |  |  |  |  |  |  |  |  |
| Outcome: Physical activity | | **Study limitations** |  | **Inconsistency** | **Indirectness** |  | **Imprecision** |  | **Publication bias** | **Moderate/large effect size (MD)** | **Adjusted for confounders** |  |
|  | | *Studies with high risk of bias* | *Prospective studies* | *Heterogeneity*  *(I^2^ > 50%)* | *Outcome* | *Patients* | *Total cases; studies with cases <100* | *Effect size (95% CI)* |  |  |  |  |
|  | |  |  |  |  |  |  |  |  |  |  |  |
| **Revision TKA: LEAS + UCLA (using crosswalk)** | | 1/16 | 5/16 ↓ | 96% ↓ | Yes ↓ | No | 1,634; 9/16 ↓ | MD 1.17 (95% CI 0.60 – 1.73) | No | 1.17 ↑ | 0/16 | Very low |
| **Revision TKA: prospective studies** | | 0/6 | 6/6 | 88% ↓ | Yes ↓ | No | 1,027; 1/6 | MD 0.89 (95% CI 0.48 – 1.30) | No | 0.89 | 0/6 | Low |
|  | |  |  |  |  |  |  |  |  |  |  |  |
|  | ↓ Downgraded quality of evidence ↑ Upgraded quality of evidence *MD* Mean difference *NR* not reported *CI* Confidence interval | | | | | | | | | | | |
