# Supplementary material for: Physical activity after revision knee arthroplasty including return to sport and work: a systematic review and meta-analysis including GRADE
Source: BMC Musculoskelet Disord. 2023 May 9;24:368. doi: 10.1186/s12891-023-06458-y (PMC10170708; doi:10.1186/s12891-023-06458-y)
Supplement: Supplementary file 4 — Additional file 4. Forest plot of meta-analysis: physical outcome measures for retrospective studies. [file 12891_2023_6458_MOESM4_ESM.docx]

*CI* Confidence Interval *IV* Inverse variance *SD* Standard deviation
