# Supplementary material for: Physical activity after revision knee arthroplasty including return to sport and work: a systematic review and meta-analysis including GRADE
Source: BMC Musculoskelet Disord. 2023 May 9;24:368. doi: 10.1186/s12891-023-06458-y (PMC10170708; doi:10.1186/s12891-023-06458-y)
Supplement: Supplementary file 5 — Additional file 5. Studies and reasons for exclusion following full text screening. [file 12891_2023_6458_MOESM5_ESM.docx]

| **Study** | **Reason for exclusion** |
| --- | --- |
| Barrack et al. [1] 2000 | Wrong outcomes |
| Meek et al. [2] 2003 | Wrong outcomes |
| Meek et al. [3] 2003 | Wrong outcomes |
| Utting et al. [4] 2004 | Wrong outcomes |
| Becker et al. [5] 2004 | Wrong outcomes |
| Jones et al. [6] 2004 | Wrong outcomes |
| Meek et al. [7] 2004 | Wrong outcomes |
| Masri et al. [8] 2006 | Wrong outcomes |
| Mulhall et al. [9] 2007 | Wrong outcomes |
| Kim et al. [10] 2009 | Wrong outcomes |
| Laudermilch et al. [11] 2010 | Wrong outcomes |
| Lavernia et al. [12] 2011 | Wrong outcomes |
| Hanna et al. [13] 2011 | Wrong outcomes |
| Malviya et al. [14] 2012 | Wrong outcomes |
| Chou et al. [15] 2012 | Wrong outcomes |
| Malviya et al. [16] 2012 | Wrong outcomes |
| Agarwal et al. [17] 2013 | Wrong outcomes |
| Venkataramanan et al. [18] 2013 | Wrong outcomes |
| Tay et al. [19] 2013 | Wrong outcomes |
| Robb et al. [20] 2013 | Wrong outcomes |
| Rao et al. [21] 2013 | Wrong outcomes |
| Kerens et al. [22] 2013 | Wrong outcomes |
| Joo et al. [23] 2013 | Wrong outcomes |
| Baier et al. [24] 2013 | Wrong outcomes |
| Pietschmann et al. [25] 2014 | Wrong outcomes |
| Luque et al. [26] 2014 | Wrong outcomes |
| Epinette et al. [27] 2014 | Wrong outcomes |
| Huang et al. [28] 2014 | Wrong outcomes |
| Kim et al. [29] 2015 | Wrong outcomes |
| Seon et al. [30] 2016 | Wrong outcomes |
| Donaldson et al. [31] 2016 | Wrong outcomes |
| Leta et al. [32] 2016 | Wrong outcomes |
| Somford et al. [33] 2016 | Wrong outcomes |
| Rajgopal et al. [34] 2017 | Wrong outcomes |
| Hamilton et al. [35] 2017 | Wrong outcomes |
| Murgier et al. [36] 2017 | Wrong outcomes |
| Scott et al. [37] 2018 | Wrong outcomes |
| Gomez-Vallejo et al. [38] 2018 | Wrong outcomes |
| Heyberger et al. [39] 2018 | Wrong outcomes |
| Lombardi et al. [40] 2018 | Wrong outcomes |
| Tsai et al. [41] 2018 | Wrong outcomes |
| Sisko et al. [42] 2019 | Wrong outcomes |
| Stockwell et al. [43] 2019 | Wrong outcomes |
| Verbeek et al. [44] 2019  Lim et al. [45] 2019 | Wrong outcomes  Wrong outcomes |
| Stevens et al. [46] 2019 | Wrong outcomes |
| Konrads et al. [47] 2019 | Wrong outcomes |
| Weissenberger et al. [48] 2020 | Wrong outcomes |
| Oliver et al. [49] 2020 | Wrong outcomes |
| Cherny et al. [50] 2021 | Wrong outcomes |
| Shelton et al. [51] 2021 | Wrong outcomes |
| Lee et al. [52] 2021 | Wrong outcomes |
| Tracey et al. [53] 2021 | Wrong outcomes |
| Baek et al. [54] 2021 | Wrong outcomes |
| Tarabichi et al. [55] 2022 | Wrong outcomes |

1. Barrack RL, Engh G, Rorabeck C, Sawhney J, Woolfrey M. Patient satisfaction and outcome after septic versus aseptic revision total knee arthroplasty. J Arthroplasty. 2000 Dec;15(8):990-3.
2. Meek RM, Masri BA, Dunlop D, Garbuz DS, Greidanus NV, McGraw R, Duncan CP. Patient satisfaction and functional status after treatment of infection at the site of a total knee arthroplasty with use of the PROSTALAC articulating spacer. J Bone Joint Surg Am. 2003 Oct;85(10):1888-92.
3. Meek RM, Greidanus NV, McGraw RW, Masri BA. The extensile rectus snip exposure in revision of total knee arthroplasty. J Bone Joint Surg Br. 2003 Nov;85(8):1120-2.
4. Utting MR, Newman JH. Customised hinged knee replacements as a salvage procedure for failed total knee arthroplasty. Knee. 2004 Dec;11(6):475-9.
5. Becker R, John M, Neumann WH. Clinical outcomes in the revision of unicondylar arthoplasties to bicondylar arthroplasties. A matched-pair study. Arch Orthop Trauma Surg. 2004 Dec;124(10):702-7.
6. Jones DL, Cauley JA, Kriska AM, Wisniewski SR, Irrgang JJ, Heck DA, Kwoh CK, Crossett LS. Physical activity and risk of revision total knee arthroplasty in individuals with knee osteoarthritis: a matched case-control study. J Rheumatol. 2004 Jul;31(7):1384-90.
7. Meek RM, Dunlop D, Garbuz DS, McGraw R, Greidanus NV, Masri BA. Patient satisfaction and functional status after aseptic versus septic revision total knee arthroplasty using the PROSTALAC articulating spacer. J Arthroplasty. 2004 Oct;19(7):874-9.
8. Masri BA, Meek RM, Greidanus NV, Garbuz DS. Effect of retaining a patellar prosthesis on pain, functional, and satisfaction outcomes after revision total knee arthroplasty. J Arthroplasty. 2006 Dec;21(8):1169-74.
9. Mulhall KJ, Ghomrawi HM, Mihalko W, Cui Q, Saleh KJ. Adverse effects of increased body mass index and weight on survivorship of total knee arthroplasty and subsequent outcomes of revision TKA. J Knee Surg. 2007 Jul;20(3):199-204.
10. Kim YH, Kim JS. Revision total knee arthroplasty with use of a constrained condylar knee prosthesis. J Bone Joint Surg Am. 2009 Jun;91(6):1440-7.
11. Laudermilch DJ, Fedorka CJ, Heyl A, Rao N, McGough RL. Outcomes of revision total knee arthroplasty after methicillin-resistant Staphylococcus aureus infection. Clin Orthop Relat Res. 2010 Aug;468(8):2067-73.
12. Lavernia C, Contreras JS, Alcerro JC. The peel in total knee revision: exposure in the difficult knee. Clin Orthop Relat Res. 2011 Jan;469(1):146-53.
13. Hanna SA, Aston WJ, de Roeck NJ, Gough-Palmer A, Powles DP. Cementless revision TKA with bone grafting of osseous defects restores bone stock with a low revision rate at 4 to 10 years. Clin Orthop Relat Res. 2011 Nov;469(11):3164-71.
14. Malviya A, Bettinson K, Kurtz SM, Deehan DJ. When do patient-reported assessments peak after revision knee arthroplasty? Clin Orthop Relat Res. 2012 Jun;470(6):1728-34.
15. Chou DT, Swamy GN, Lewis JR, Badhe NP. Revision of failed unicompartmental knee replacement to total knee replacement. Knee. 2012 Aug;19(4):356-9.
16. Malviya A, Brewster NT, Bettinson K, Holland JP, Weir DJ, Deehan DJ. Functional outcome following aseptic single-stage revision knee arthroplasty. Knee Surg Sports Traumatol Arthrosc. 2012 Oct;20(10):1994-2001.
17. Agarwal S, Azam A, Morgan-Jones R. Metal metaphyseal sleeves in revision total knee replacement. Bone Joint J. 2013 Dec;95-B(12):1640-4.
18. Venkataramanan V, Gignac MA, Dunbar M, Garbuz D, Gollish J, Gross A, Hedden D, MacDonald SJ, Mahomed NN, Schemitsch E, Davis AM. The importance of perceived helplessness and emotional health in understanding the relationship among pain, function, and satisfaction following revision knee replacement surgery. Osteoarthritis Cartilage. 2013 Jul;21(7):911-7.
19. Tay KS, Lo NN, Yeo SJ, Chia SL, Tay DK, Chin PL. Revision total knee arthroplasty: causes and outcomes. Ann Acad Med Singap. 2013 Apr;42(4):178-83.
20. Robb CA, Matharu GS, Baloch K, Pynsent PB. Revision surgery for failed unicompartmental knee replacement: technical aspects and clinical outcome. Acta Orthop Belg. 2013 Jun;79(3):312-7.
21. Rao BM, Kamal TT, Vafaye J, Moss M. Tantalum cones for major osteolysis in revision knee replacement. Bone Joint J. 2013 Aug;95-B(8):1069-74.
22. Kerens B, Boonen B, Schotanus MG, Lacroix H, Emans PJ, Kort NP. Revision from unicompartmental to total knee replacement: the clinical outcome depends on reason for revision. Bone Joint J. 2013 Sep;95-B(9):1204-8.
23. Joo JH, Lee SC, Ahn NK, Ahn HS, Jung KA. Patellar resurfacing versus no resurfacing in two-stage revision of infected total knee arthroplasty. Knee. 2013 Dec;20(6):451-6.
24. Baier C, Lüring C, Schaumburger J, Köck F, Beckmann J, Tingart M, Zeman F, Grifka J, Springorum HR. Assessing patient-oriented results after revision total knee arthroplasty. J Orthop Sci. 2013 Nov;18(6):955-61.
25. Pietschmann MF, Ficklscherer A, Wohlleb L, Schmidutz F, Jansson V, Müller PE. UKA can be safely revised to primary knee arthroplasty by using an autologous bone plate from the proximal lateral tibia. J Arthroplasty. 2014 Oct;29(10):1991-5.
26. Luque R, Rizo B, Urda A, Garcia-Crespo R, Moro E, Marco F, López-Duran L. Predictive factors for failure after total knee replacement revision. Int Orthop. 2014 Feb;38(2):429-35.
27. Epinette JA, Leyder M, Saragaglia D, Pasquier G, Deschamps G; Société Française de la Hanche et du Genou. Is unicompartmental-to-unicompartmental revision knee arthroplasty a reliable option? Case-control study. Orthop Traumatol Surg Res. 2014 Feb;100(1):141-5.
28. Huang R, Barrazueta G, Ong A, Orozco F, Jafari M, Coyle C, Austin M. Revision total knee arthroplasty using metaphyseal sleeves at short-term follow-up. Orthopedics. 2014 Sep;37(9):e804-9.
29. Kim YH, Park JW, Kim JS, Oh HK. Long-Term Clinical Outcomes and Survivorship of Revision Total Knee Arthroplasty with Use of a Constrained Condylar Knee Prosthesis. J Arthroplasty. 2015 Oct;30(10):1804-9.
30. Seon JK, Song EK. Joint line and patellar height restoration after revision total knee arthroplasty. Indian J Orthop. 2016 Mar-Apr;50(2):159-65.
31. Donaldson JR, Tudor F, Gollish J. Revision surgery for the stiff total knee arthroplasty. Bone Joint J. 2016 May;98-B(5):622-7.
32. Leta TH, Lygre SH, Skredderstuen A, Hallan G, Gjertsen JE, Rokne B, Furnes O. Outcomes of Unicompartmental Knee Arthroplasty After Aseptic Revision to Total Knee Arthroplasty: A Comparative Study of 768 TKAs and 578 UKAs Revised to TKAs from the Norwegian Arthroplasty Register (1994 to 2011). J Bone Joint Surg Am. 2016 Mar 16;98(6):431-40.
33. Somford MP, Brouwer RW, Haen PW, van Raay JJ, van Raaij TM. Technical aspects of revision and functional outcome after revision of the Oxford unicompartmental knee arthroplasty. Knee. 2016 Dec;23(6):1020-1023.
34. Rajgopal A, Panjwani TR, Rao A, Dahiya V. Are the Outcomes of Revision Knee Arthroplasty for Flexion Instability the Same as for Other Major Failure Mechanisms? J Arthroplasty. 2017 Oct;32(10):3093-3097.
35. Rajgopal A, Panjwani TR, Rao A, Dahiya V. Are the Outcomes of Revision Knee Arthroplasty for Flexion Instability the Same as for Other Major Failure Mechanisms? J Arthroplasty. 2017 Oct;32(10):3093-3097.
36. Murgier J, Cailliez J, Wargny M, Chiron P, Cavaignac E, Laffosse JM. Cryotherapy With Dynamic Intermittent Compression Improves Recovery From Revision Total Knee Arthroplasty. J Arthroplasty. 2017 Sep;32(9):2788-2791.
37. Scott CEH, Powell-Bowns MFR, MacDonald DJ, Simpson PM, Wade FA. Revision of Unicompartmental to Total Knee Arthroplasty: Does the Unicompartmental Implant (Metal-Backed vs All-Polyethylene) Impact the Total Knee Arthroplasty? J Arthroplasty. 2018 Jul;33(7):2203-2209.
38. Gómez-Vallejo J, Albareda-Albareda J, Seral-García B, Blanco-Rubio N, Ezquerra-Herrando L. Revision total knee arthroplasty: hybrid vs standard cemented fixation. J Orthop Traumatol. 2018 Aug 17;19(1):9.
39. Heyberger C, Auberger G, Babinet A, Anract P, Biau DJ. Patients with Revision Modern Megaprostheses of the Distal Femur Have Improved Disease-Specific and Health-Related Outcomes Compared to Those with Primary Replacements. J Knee Surg. 2018 Oct;31(9):822-826.
40. Lombardi AV Jr, Kolich MT, Berend KR, Morris MJ, Crawford DA, Adams JB. Revision of Unicompartmental Knee Arthroplasty to Total Knee Arthroplasty: Is It as Good as a Primary Result? J Arthroplasty. 2018 Jul;33(7S):S105-S108.
41. Tsai SW, Chen CF, Wu PK, Chen CM, Chen WM. Revision Total Knee Arthroplasty Using a Constrained Condylar Knee Prosthesis Combined with a Posterior Stabilized Articular Surface. J Knee Surg. 2018 Feb;31(2):197-201.
42. Sisko ZW, Vasarhelyi EM, Somerville LE, Naudie DD, MacDonald SJ, McCalden RW. Morbid Obesity in Revision Total Knee Arthroplasty: A Significant Risk Factor for Re-Operation. J Arthroplasty. 2019 May;34(5):932-938.
43. Stockwell KD, Malleck S, Gascoyne TC, Turgeon TR. Clinical and radiographic outcomes of a hybrid fixation revision total knee arthroplasty system at short to mid-term follow-up. Knee. 2019 Jan;26(1):240-249.
44. Verbeek JFM, Hannink G, Defoort KC, Wymenga AB, Heesterbeek PJC. Age, gender, functional KSS, reason for revision and type of bone defect predict functional outcome 5 years after revision total knee arthroplasty: a multivariable prediction model. Knee Surg Sports Traumatol Arthrosc. 2019 Jul;27(7):2289-2296.
45. Lim JBT, Pang HN, Tay KJD, Chia SL, Lo NN, Yeo SJ. Clinical outcomes and patient satisfaction following revision of failed unicompartmental knee arthroplasty to total knee arthroplasty are as good as a primary total knee arthroplasty. Knee. 2019 Aug;26(4):847-852.
46. Stevens JM, Clement ND, MacDonald D, Hamilton DF, Burnett R. Survival and functional outcome of revision total knee arthroplasty with a total stabilizer knee system: minimum 5 years of follow-up. Eur J Orthop Surg Traumatol. 2019 Oct;29(7):1511-1517.
47. Konrads C, Franz A, Hoberg M, Rudert M. Similar Outcomes of Two-Stage Revisions for Infection and One-Stage Revisions for Aseptic Revisions of Knee Endoprostheses. J Knee Surg. 2019 Sep;32(9):897-899.
48. Weißenberger M, Petersen N, Bölch S, Rak D, Arnholdt J, Rudert M, Holzapfel BM. Revision of unicompartmental knee arthroplasty using the in situ referencing technique. Oper Orthop Traumatol. 2020 Aug;32(4):273-283.
49. Oliver G, Jaldin L, Camprubí E, Cortés G. Observational Study of Total Knee Arthroplasty in Aseptic Revision Surgery: Clinical Results. Orthop Surg. 2020 Feb;12(1):177-183.
50. Cherny AA, Kovalenko AN, Kulyaba TA, Kornilov NN. A prospective study on outcome of patient-specific cones in revision knee arthroplasty. Arch Orthop Trauma Surg. 2021 Dec;141(12):2277-2286.
51. Shelton TJ, Gill M, Athwal G, Howell SM, Hull ML. Revision of a Medial UKA to a Kinematic Aligned TKA: Comparison of Operative Complexity, Postoperative Alignment, and Outcome Scores to a Primary TKA. J Knee Surg. 2021 Mar;34(4):406-414.
52. Lee SS, Park JS, Lee YK, Moon YW. Comparison of the clinical and radiological outcomes between an isolated tibial component revision and total revision knee arthroplasty in aseptic loosening of an isolated tibial component. J Orthop Sci. 2021 May;26(3):435-440.
53. Tracey RW, Akram F, Della Valle CJ, Sporer SM, Berger RA, Gerlinger TL. Clinical Outcomes in Isolated Tibial Revision With Cruciate Retaining Total Knee Arthroplasty. J Arthroplasty. 2021 Jul;36(7):2536-2540.
54. Baek JH, Lee SC, Jin H, Kim JW, Ahn HS, Nam CH. Poor outcomes of revision total knee arthroplasty in patients with septic loosening compared to patients with aseptic loosening. J Orthop Surg Res. 2021 Oct 18;16(1):624.
55. Tarabichi S, Grau L, Arshi A, Post Z, Ong A, Hozack WJ. Clinical and Radiographic Outcomes of Novel 3D-Printed Highly Porous Knee Cone Design. Surg Technol Int. 2022 Feb 24;40:sti40/1563.
